# Three-State Majority-vote Model on Scale-Free Networks and the Unitary Relation for Critical Exponents

**DOI:** 10.1038/s41598-020-63929-1

**Published:** 2020-05-19

**Authors:** André L. M. Vilela, Bernardo J. Zubillaga, Chao Wang, Minggang Wang, Ruijin Du, H. Eugene Stanley

**Affiliations:** 1 Física de Materiais, Universidade de Pernambuco, 50720-001 Recife, Pernambuco Brazil; 2 Center for Polymer Studies and Department of Physics, Boston University, Boston, Massachusetts 02215 USA; 3 College of Economics and Management, Beijing University of Technology, Beijing, 100124 China; 4 School of Mathematical Science, Nanjing Normal University, Nanjing, 210042 Jiangsu China; 5 Department of Mathematics, Nanjing Normal University Taizhou College, Taizhou, 225300 Jiangsu China; 6 Institute of Applied System Analysis, Jiangsu University, Zhenjiang, 212013 Jiangsu China

**Keywords:** Complex networks, Phase transitions and critical phenomena

## Abstract

We investigate the three-state majority-vote model for opinion dynamics on scale-free and regular networks. In this model, an individual selects an opinion equal to the opinion of the majority of its neighbors with probability 1 − *q*, and different to it with probability *q*. The parameter *q* is called the noise parameter of the model. We build a network of interactions where *z* neighbors are selected by each added site in the system, a preferential attachment network with degree distribution *k*^−*λ*^, where *λ* = 3 for a large number of nodes *N*. In this work, *z* is called the growth parameter. Using finite-size scaling analysis, we obtain that the critical exponents $\beta /\bar{\nu }$ and $\gamma /\bar{\nu }$ associated with the magnetization and the susceptibility, respectively. Using Monte Carlo simulations, we calculate the critical noise parameter *q*_*c*_ as a function of *z* for the scale-free networks and obtain the phase diagram of the model. We find that the critical exponents add up to unity when using a special volumetric scaling, regardless of the dimension of the network of interactions. We verify this result by obtaining the critical noise and the critical exponents for the two and three-state majority-vote model on cubic lattice networks.

## Introduction

**Figure 1:**
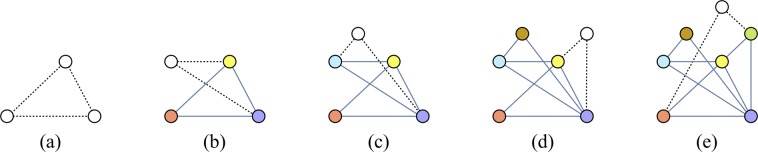
The sequence shows five subsequent steps of the Barabási-Albert model for the growth of scale-free networks with *z* = 2. Empty circles mark the newly added node to the network, and dashed lines represent its new links, which connect using the preferential attachment.

Regular networks and random graphs are widely used to study and describe the structure of diverse systems investigated in condensed matter physics. Still, they do not capture several behaviors of real networks found in nature^1–6^. By using the complex network framework, scientists studied a wide variety of physical systems such as the world wide web, cellular networks, protein-protein interaction networks, the scientific collaboration network, airline networks, economic and financial markets, among others^7–16^. Many real systems are labeled by networks that present the same universal features, or the same architectures of assembly. One of the most investigated kinds of networks present in real-world systems are the scale-free networks^1–3^. These networks can be built using the Barabási-Albert model. In this model, two simple mechanisms - growth and preferential attachment - are responsible for the emergence of scale-free networks. By starting with an initial number of interconnected nodes (or core), one adds new nodes that have a higher probability of attaching to the more connected nodes already present in a mechanism known as preferential attachment. In this process, highly connected nodes acquire more links to other nodes than those that have fewer connections, yielding sites with a very high number of neighbors. These sites are called the hubs of the network. The degree distribution of the scale-free networks built by the Barabási-Albert model presents a power-law decay with exponent *λ* = 3 for a large number of nodes *N*. In Fig. 1 we illustrate the preferential attachment algorithm for a network where each newly added site connects to others with the growth parameter *z* = 2.

The scale-free distribution and its mathematical structure have motivated several studies investigating the phase transition of different ferromagnetic spin systems^17–20^. For the Ising model, the increase of the temperature induces the system to be in a fully disordered state, where all the possible configurations are accessible due to the high thermal energy available. In these works, the authors obtain a linear dependence between the critical temperature *T*_*c*_, and the average number of interacting spins 〈*k*〉 for the scale-free networks investigated^17^. They also find that the critical temperature of the Ising model is proportional to the logarithm of the finite system size *N*^18^. This result was also verified for the Potts model with two states on semi-directed Barabási-Albert networks^20^.

The complex network framework allowed physicists to propose models aiming to investigate the critical phenomena in social dynamics^21–35^. Although human individuals may be difficult to simulate, the dynamics of people in groups seem to be conceivable to model due to herd effects and other group behaviors. By employing statistical physics techniques, several works confirm that these models present order-disorder transitions and universality among other standard characteristics of condensed matter physical systems. From microscopic interactions among individuals in a social network, several sociophysics models exhibit a robust collective behavior. Concerning opinion dynamics in complex networks, we highlight the majority-vote model with noise^24^. In this model, an individual assumes the same opinion of the majority of its neighbors with probability *p* and the opposite opinion with probability *q* = (1 − *p*). In its two-state version, the opinion of an individual is represented by the spin variable *σ*, which assumes the value +1 or −1 in a given time. In a regular square lattice of interactions, this model undergoes a nonequilibrium phase transition at a critical probability ${q}_{c}\simeq 0.075$, and the critical exponents are the same as those of the equilibrium two-dimensional magnetic Ising model.

The three-state majority-vote model with noise is a system of spins, where each one is allowed to be in one of three states, that is *σ* = 1, 2, or 3^31–33^. As in the two-state model, each spin assumes the state of the majority of its neighboring spins with probability *p* = (1 − *q*) and the opposite state with probability *q*, which is known as the noise parameter of the model. The increase of the parameter *q* promotes the formation of different opinion configurations in the model, acting as an increase of the social temperature of the system. For a regular square lattice network, the three-state majority-vote model with noise present an order-disorder phase transition at the critical noise value ${q}_{c}\simeq 0.118$, where the consensus in the system vanishes.

In this work, we investigate the influence of a network with preferential attachment, built by using the classical Barabási-Albert model, on the three-state majority-vote model with noise. We perform Monte Carlo simulations to estimate the critical noise parameter as a function of the growth parameter *q*_*c*_(*z*). Moreover, we use the standard finite-size scaling techniques to obtain the critical exponents for several values of the parameter *z* for the networks investigated. Based on the results, we propose a unitary relation to examine the criticality of the system. We conjecture that this relation is universal, regardless of the network of interactions. We also perform simulations for the three-state majority-vote model on cubic networks that confirm our results, and we obtain the critical noise for this system and its critical exponents.

The remainder of this paper is divided into four sections. In Section II, we describe the three-state majority-vote model with noise for opinion dynamics, the network construction process, and introduce the relevant quantities used in our simulations. Section III contains our results for complex and regular networks, along with a discussion. In Section IV, we present our conclusions and final remarks.

## The Model

### The Barabási-Albert network

The three-state majority-vote model with noise consists in a set of spin variables {σ_*i*_} with *i* = 1, 2, …, *N*, where each variable can be assigned to one of the values *σ* = 1, 2, or 3, representing the opinion for an individual in the community at a given time *t*. The individuals are distributed in the nodes of a scale-free network of social interactions with *N* sites. That is, before adding individuals and their opinions in our system, we build a network of interactions. We start with a core of *z* +1  fully connected nodes, and then we add new nodes - one at a time - with *z* free links, which will be connected by preferential attachment to the existing nodes of the network. The probability of connecting a new node *j* to node *i*, $\Pi ({k}_{i})$, depends on the degree *k*_*i*_ of the node *i*. Thus, for Barabási-Albert networks with linear preferential attachment we write

1 $$\Pi ({k}_{i})=\frac{{k}_{i}}{\sum _{\ell }\,{k}_{\ell }},$$

where the summation is equal to the total number of existing links in the scale-free network, and more nodes are added to the network until it reaches a total of *N* sites. A double connection to the same site is forbidden.

**Figure 2:**
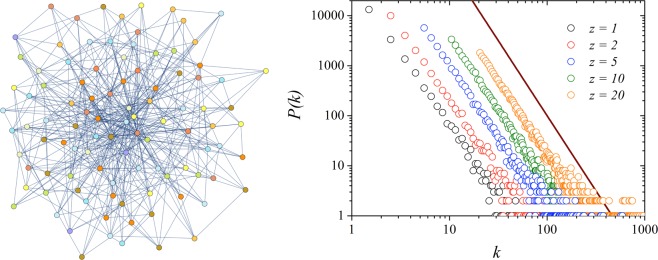
A illustration of the Barabási-Albert network with *N* = 100 nodes and *z* = 5 (left), and the degree distribution histogram *P*(*k*) for a single network of size *N* = 20000, with *z* = 1, 2, 5, 10 and 20 (right). The straight line is a guide to the eye and has slope corresponding to the network’s predicted degree exponent of decay *λ* = 3.

In Fig. 2 we show an illustration of the Barabási-Albert network with *N* = 100 sites for *z* = 5 (left). Observe that some nodes have a high number of connections, despite the small average value of links per site in the network. We also present the histogram of the degree of the nodes for networks with *N* = 20000 nodes and different values of the growth parameter *z* (right), where we obtain the characteristic scale-free degree distribution plot with exponent *λ* = 3. Note that the decay exponent *λ* is the same, even for different values of the average degree per node *z* as expected^2^.

### Dynamics and numerical quantities

The dynamics of the system consists of a generalization for three opinions of the two-state majority-vote model^24–26,28–33,36–43^. For a randomly selected individual *σ*_*i*_ we determine the majority opinion of the individuals that are linked to it. With probability 1 − *q*, the selected agent *σ*_*i*_ adopts the dominant opinion of its neighbors (follows the majority), and with probability *q*, the individual adopts a different opinion (follow a minority). For a tie between the three states, the selected agent *σ*_*i*_ changes to any opinion with the same probability equal to 1/3. For the case of a tie between two majorities, *σ*_*i*_ assumes one of these tied states with probability (1 − *q*)/2, and the minority with probability *q*. Finally, for the case of a single majority, *σ*_*i*_ follows any of the two minorities with probability *q*/2, and the majority with probability 1 − *q*. That is, if *n*_*α*_ is the number of neighbors of the individual *σ*_*i*_ in a given state *α* = 1, 2, 3, then, the probabilities for *σ*_*i*_ to assume the opinion *α* = 1 is:

2 $$\begin{array}{c}P(1|{n}_{1} > {n}_{2},{n}_{3})=1-q,\,P(1|{n}_{1}={n}_{2} > {n}_{3})=(1-q)/2,\,P(1|{n}_{1} < {n}_{2}={n}_{3})=q,\\ P(1|{n}_{1},{n}_{2} < {n}_{3})=q/2,\,(1|{n}_{1}={n}_{2}={n}_{3})=1/3.\end{array}$$

These transition rules present the *C*_3*v*_ symmetry for the simultaneous change of all opinions. The probabilities for the other states *σ* = 2 and 3 are obtained by symmetry operations. The total number of individuals connected to *σ*_*i*_ is *n* = *n*_1_ + *n*_2_ + *n*_3_. We remark that all probabilities satisfy the relation

3 $$P(1|\ldots )+P(2|\ldots )+P(3|\ldots )=1.$$

In our simulations, we choose the consequential update for the opinion dynamics of the agents. To investigate the critical behavior of the three-state majority-vote model, we first calculate the average opinion, defined by

4 $$m={({m}_{1}^{2}+{m}_{2}^{2}+{m}_{3}^{2})}^{1/2},$$

whose normalized components are given by

5 $${m}_{\alpha }=\sqrt{\frac{3}{2}}\left[\frac{1}{N}\mathop{\sum }\limits_{i=1}^{N}\,\delta (\alpha ,{\sigma }_{i})-\frac{1}{3}\right],$$

where the sum is over all sites in the scale-free network of social interactions and *δ*(*α*, *σ*_*i*_) is the Kronecker delta function. In this way, to investigate the critical behavior of the model, we consider the following numerical quantities: the magnetization *M*, the magnetic susceptibility *χ*, and the Binder’s fourth-order cumulant *U* defined by

6 $$M(q,z,N)={\langle {\langle m\rangle }_{t}\rangle }_{c},$$

7 $$\chi (q,z,N)=N[{\langle {\langle {m}^{2}\rangle }_{t}\rangle }_{c}-{\langle {\langle m\rangle }_{t}\rangle }_{c}^{2}],$$

8 $$U(q,z,N)=1-\frac{{\langle {\langle {m}^{4}\rangle }_{t}\rangle }_{c}}{3{\langle {\langle {m}^{2}\rangle }_{t}\rangle }_{c}^{2}},$$

where *q* is the noise parameter, *z* is the growth parameter of the network, *N* is the total number of individuals or agents, 〈…〉_*t*_ represents the time average taken in the stationary regime and 〈…〉_*c*_ denotes the configurational average. The critical behavior of the model is investigated by performing computer simulations and by using finite-size scaling analysis.

The three-state majority-vote dynamics evolves over time according to the probability rules given by Eq. (2). After a transient, the model reaches a steady state that presents complete order, partial order(disorder) or complete disorder. That is, for *q* = 0 the system exhibits an ordered steady macrostate, characterized by the predominance of individuals with one of the three possible opinions. Assuming for this case that *σ* = 1 ∀ *N*, we obtain ${m}_{1}=\sqrt{2/3}$, ${m}_{2}={m}_{3}=-1/\sqrt{6}$ and *m* = 1, yielding *M* = 1 for *q* = 0. The upper limit for *q*, that is, the infinite social temperature, is obtained when the probability of agreeing with the majority is equal to the probability of agreeing with any minority, thus $1-q=q/2\Rightarrow q=2/3$. In this case, any opinion or individual state *σ* = 1, 2 or 3 can be found with equal probability in the network of social interactions. Thus, leading to ${m}_{1},{m}_{2},{m}_{3}\simeq 0$ and *M*(*q* = 2/3) = 0 in the thermodynamic limit *N* → ∞.

## Discussion and Results

### Monte Carlo simulations

We perform numerical simulations on scale-free networks with sizes ranging from *N* = 1000 to 20000 using the Monte Carlo method. For each pair of values for the parameters *q* and *z*, we set a fraction of sites *f*_0_ = 0.8 to point to one opinion, i.e., *σ*_*i*_ = 1, and the remaining fraction 1 − *f*_0_ to point to the other two options, equally distributed. We next select a randomly chosen individual and update its opinion with the probabilities given by Eq. (2). This process is repeated *N* times to allow that the opinion of each agent is updated once (on average) for each Monte Carlo Step (MCS). We skip 10^5^ MCS in the simulation to overcome the initial transients and allow the system to reach a steady-state, characterized by the set of parameters *q* and *z*. Next, we perform the time averages in the following 2 × 10^5^ MCS. For each realization, we generate at least 100 independent random samples to obtain the configurational averages. Different values for *f*_0_ yield the same final steady-state for the system, which becomes ordered or disordered, depending on the values for *q*, *z*, and *N*. In the ordered phase, the dominant opinion is found in one of the possible states 1, 2, or 3. In the disordered phase, the three opinions are equally distributed in the network of social interactions.

**Figure 3:**
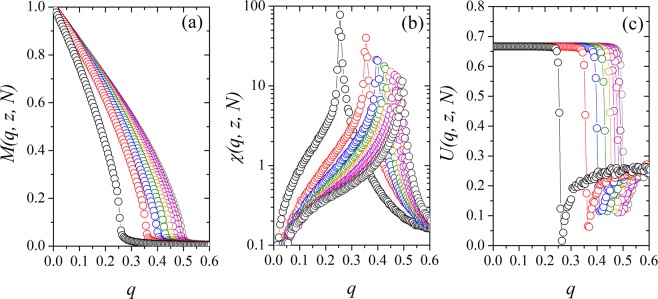
Three-state majority-vote model on Barabási-Albert networks for *N* = 20000. (**a**) Magnetization *M*(*q*, *z*, *N*), (**b**) susceptibility *χ*(*q*, *z*, *N*) and (**c**) Binder’s fourth-order cumulant *U*(*q*, *z*, *N*) as a function of the noise parameter *q* for *z* = 2, 3, 4, 5, …, 10, from left to right. The error bars are smaller than the symbol size, and the lines are just a guide to the eyes.

In Fig. 3, we illustrate the effect of the network of interactions with preferential attachment in the consensus (order) of the system. We show the plot for the magnetization *M*(*q*, *z*, *N*), for the susceptibility *χ*(*q*, *z*, *N*) and for the Binder’s fourth-order cumulant *U*(*q*, *z*, *N*) as a function of the noise *q* for *N* = 20000 and *z* = 2, 3, 4, 5, …, 10. We find that for small values of the noise parameter *q*, the system presents an ordered macrostate, or phase, where *M*(*q*, *z*, *N*) = *O*(1). In this phase, the society becomes ordered, with a preference for a dominant opinion. By increasing the social temperature *q*, the magnetization will continuously decrease to zero near a critical value *q*_*c*_, denoting the second-order phase transition of the system. In this region (*q* > *q*_*c*_), there is no prevailing opinion in the society, and every state can be found with the same probability. In Fig. 3(b) the magnetic susceptibility *χ*(*q*, *z*, *N*) exhibits a maximum near some critical value *q*_*c*_, where the transition order-disorder occurs. This behavior is also denoted by the rapid decrease of the Binder’s fourth-order cumulant *U*(*q*, *z*, *N*), showed in Fig. 3(c). From the results, we obtain that the critical noise is an increasing function of the growth parameter of the network *z*, indicating that the consensus is stronger if there are more connections to an individual in the society.

**Figure 4:**
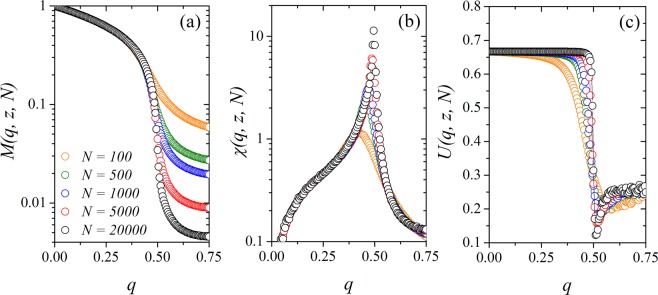
(**a**) Magnetization *M*(*q*, *z*, *N*), (**b**) susceptibility *χ*(*q*, *z*, *N*) and (**c**) Binder’s fourth-order cumulant as a function of the noise parameter *q* for several values of the system size *N* with *z* = 10. The critical noise for this value of the growth parameter is *q*_*c*_ = 0.513(1), obtained at the intersection point for Binder cumulant (**c**). The error bars are smaller than the symbol size, and the line is a guide to the eye.

Next, we consider the finite-size effects on our measured quantities. In Fig. 4 we show the (a) magnetization *M*(*q*, *z*, *N*), (b) the susceptibility *χ*(*q*, *z*, *N*) and (c) the Binder’s fourth-order cumulant *U*(*q*, *z*, *N*) versus the noise parameter *q* for *z* = 10, and several system sizes *N*. We remark that *M*(*q*, *z*, *N*) ≠ 0 for high values of the parameter *q* due to finite-size effects. The susceptibility *χ*(*q*, *z*, *N*) exhibits a sharper peak as we increase the system size, and the position of its maximum in the horizontal axis depends on *N*. Thus, we write the pseudocritical noise as *q*_*c*_(*z*, *N*), and the values for *q*_*c*_(*z*, *N*) are our first estimative for the critical noises of the system. To obtain the critical noise for each *z* in the thermodynamic limit, *q*_*c*_(*z*), we calculate the Binder cumulant of the system *U*(*q*, *z*, *N*). In Fig. 4(c) we show the Binder’s fourth-order cumulant as a function of the social temperature *q* for different system sizes. The critical noise parameter *q*_*c*_(*z*) can be estimated for the value of *q* where the curves of *U*(*q*, *z*, *N*) for different system sizes intercept each other. In this figure, we estimate *q*_*c*_ = 0.513 (1).

**Figure 5:**
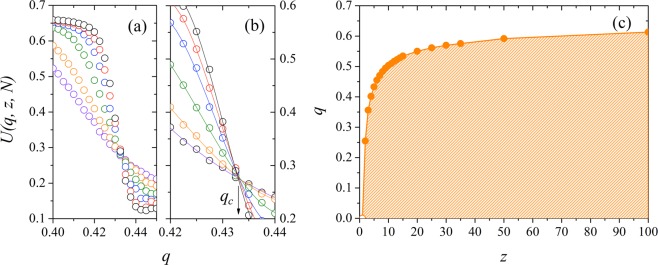
(**a**) The Binder’s fourth-order cumulant *U*(*q*, *z*, *N*) as a function of the noise parameter *q* for the three-state majority-vote model in Barabási-Albert networks with *z* = 5 for several values of the system sizes. From top to bottom we have *N* = 20000, 15000, 10000, 5000, 2000 and 1000. In (**b**), we exhibit the details of the interception for different system sizes and a cubic fit for the data points. Within the accuracy of the data, all curves intersect at *q*_*c*_ = 0.4326(4). (**c**) Phase diagram of the three-state majority-vote model on Barabási-Albert networks. The orange region denotes the phase where the system presents an order or a global majority opinion. The solid line is just a guide to the eye. In all plots, the error bars are smaller than the symbol size.

Figure 5(a) illustrates the dependence of the Binder’s fourth-order cumulant on the noise *q* for *z* = 5 and different system sizes. The curves for different values of *N* intercept in the region 0.43 < *q* < 0.44. Figure 5(b) shows the details of the Binder cumulant data, along with a polynomial fit for the lines near the interception. At this point, the critical noise does not depend on the system size, and for this case, shown in figure *q*_*c*_ = 0.4326 (4). By calculating the cumulant *U*(*q*, *z*, *N*) for other values of *z*, we obtain the phase diagram for the three-state majority-vote model on Barabási-Albert scale-free networks shown in Fig. 5(c). The orange region denotes the ordered phase of the system, where one of the three opinions is the majority state of the system. In this result, the error bars are smaller than the thickness of the line. Note that the critical noise increases with the growth parameter *z*, which controls the average number of connections 〈*k*〉 of a given individual in the social system. Here, we obtain *q*_*c*_ = 2/3 as *z* → ∞, where *q*_*c*_ = 2/3 is the infinite temperature equivalent for the social system of the three-state majority-vote model. This result agrees with the critical temperature obtained in other studies for ferromagnetic spin systems in scale-free networks^17,18^.

### The unitary relation and scaling results

To obtain the critical exponents in complex networks, we propose that near the critical noise *q*_*c*_ the correlation length *ξ* scales with the actual volume of the system^44,45^ as

9 $$\xi \sim N.$$

Thus, the pseudocritical noise *q*_*c*_(*N*), the magnetization *M*(*q*, *z*, *N*), the susceptibility *χ*(*q*, *z*, *N*), and the Binder cumulant *U*(*q*, *z*, *N*) satisfy the finite-size scaling relations

10 $${q}_{c}(N)={q}_{c}+b{N}^{-1/\bar{\nu }},$$

11 $$M(q,z,N)={N}^{-\beta /\bar{\nu }}\tilde{M}(\varepsilon {N}^{1/\bar{\nu }}),$$

12 $$\chi (q,z,N)={N}^{\gamma /\bar{\nu }}\tilde{\chi }(\varepsilon {N}^{1/\bar{\nu }}),$$

13 $$U(q,z,N)=\tilde{U}(\varepsilon {N}^{1/\bar{\nu }}),$$

where *ε* = *q* − *q*_*c*_ is the distance to the critical noise, *b* is a constant, and $\tilde{M}$, $\tilde{\chi }$, and $\tilde{U}$ are scaling functions that only depend on the scaled variable $x=\varepsilon {N}^{1/\bar{\nu }}$. For regular networks, we recall that *N* = *L*^*d*^, where *d* is the effective dimension of the network, and *L* is an effective linear size of the system. In this case, we obtain for the magnetization and the magnetic susceptibility

14 $$M(q,z,N)={L}^{-d\beta /\bar{\nu }}\tilde{M}(\varepsilon {N}^{1/\bar{\nu }}),$$

15 $$\chi (q,z,N)={L}^{d\gamma /\bar{\nu }}\tilde{\chi }(\varepsilon {N}^{1/\bar{\nu }}).$$

We use the notation $\bar{\nu }$ instead of *v* since we changed the correlation length scaling relation from the usual linear scaling *ξ* ~ *L* to a “volumetric scaling” *ξ* ~ *L*^*d*^. In this case, the hyperscaling relation now reads $2\beta d/\bar{\nu }+\gamma d/\bar{\nu }=d$. Thus, we obtain

16 $$\frac{2\beta }{\bar{\nu }}+\frac{\gamma }{\bar{\nu }}=1,$$

regardless of the effective dimension *d* of the network. This result allows us to remark that the hyperscaling relation cannot be used to estimate the dimension of these networks when using the volumetric scaling *ξ* ~ *L*^*d*^, in contrast to the results of previous studies^25,28,33,38,39^. Nevertheless, the unitary relation (Eq. (16)) was verified in these works for random graphs and scale-free networks. In this context, we rewrite the unitary relation by denoting a new exponent upsilon $\upsilon$, defined as

17 $$\upsilon \equiv \frac{2\beta }{\bar{\nu }}+\frac{\gamma }{\bar{\nu }},$$

where we conjecture that the exponent $\upsilon =1$ for any network under the condition of the volumetric scaling of Eq. (9). In this work, we denote the equation $\upsilon =1$ as the unitary relation for critical exponents. We validate the consistency of this result according to the comparison with the numerical findings for the critical exponents $\beta /\bar{\nu }$ and $\gamma /\bar{\nu }$ for regular and complex networks.

By calculating the logarithm of Eqs. (10), (11) and (12) at the critical point *q*_*c*_, we obtain an explicit relation involving the critical exponents, the measured quantities and the system volume *N*

18 $$\mathrm{ln}[{q}_{c}(N)-{q}_{c}]\sim -\frac{1}{\bar{\nu }}\,\mathrm{ln}\,N,$$

19 $$\mathrm{ln}[M(q,z,N)]\sim -\frac{\beta }{\bar{\nu }}\,\mathrm{ln}\,N,$$

20 $${\rm{l}}{\rm{n}}[\chi (q,z,N)]\sim \frac{\gamma }{\bar{\nu }}\,{\rm{l}}{\rm{n}}\,N,$$

and we use the Eqs. (18), (19) and (20) to obtain the critical exponents of the system.

**Figure 6:**
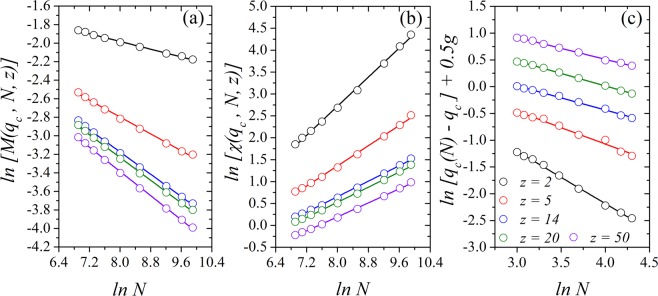
Plot at the critical point *q*_*c*_(*z*) for the logarithm of the (**a**) magnetization ln *M*(*q*_*c*_, *z*, *N*), (**b**) magnetic susceptibility ln *χ*(*q*_*c*_, *z*, *N*), and (**c**) ln [*q*_*c*_(*N*) − *q*_*c*_] versus ln *N* using *z* = 2, 5, 14, 20 and 50, with *g* = 1, 2, 3, 4 and 5. Here, *g* is an integer used to better display the data. In all plots the error bars are smaller than the symbol size.

Figure 6 shows the logarithm of the (a) magnetization, of the (b) susceptibility and of the distance between the pseudocritical noise and the critical noise $[{q}_{c}(N)-{q}_{c}]$ versus the logarithm of the volume of the system *N*, where *q* is set to be equal to *q*_*c*_(*z*). In this figure we show our results for *z* = 2, 5, 14, 20, and 50, where the angular coefficient of the lines give us an estimation of the critical exponents $1/\bar{\nu }$, $\beta /\bar{\nu }$ and $\gamma /\bar{\nu }$. We find $\beta /\bar{\nu }=0.102(4),0.229(5),0.300(3),0.307(3)$ and 0.326(4), and $\gamma /\bar{\nu }=0.83(1),0.59(1),0.44(1),0.43(1)$ and 0.40(1), and $1/\bar{\nu }=1.01(2),0.61(3),0.45(1),0.47(1)$ and 0.40(1), for *z* = 2, 5, 14, 20, and 50, respectively.

Table 1 provides critical noise, critical exponents, and the unitary relation values for each growth parameter investigated in the model. Note that the critical exponent for the magnetization (susceptibility) is a decreasing (an increasing) function of the growth parameter *z*. The critical noise *q*_*c*_ increases with *z*, while the critical exponent $1/\bar{\nu }$ decrease with *z*. For all values of the critical exponents, we obtain $\upsilon \sim 1$ as expected.

**Table 1:** The critical noise *q*_*c*_, the critical exponents $\beta /\bar{\nu }$, $\gamma /\bar{\nu }$ and $1/\bar{\nu }$, and the unitary relation $\upsilon$, for the three-state majority-vote model on Barabási-Albert networks with growth parameter *z*.

*z*	*q*_*c*_	${\boldsymbol{\beta }}{\boldsymbol{/}}\bar{{\boldsymbol{\nu }}}$	${\boldsymbol{\gamma }}{\boldsymbol{/}}\bar{{\boldsymbol{\nu }}}$	${\bf{1}}{\boldsymbol{/}}\bar{{\boldsymbol{\nu }}}$	${\boldsymbol{\upsilon }}$
2	0.2549(3)	0.102(4)	0.83(1)	1.01(2)	1.04(3)
3	0.3561(5)	0.141(4)	0.75(1)	0.82(3)	1.03(2)
4	0.4015(5)	0.197(5)	0.64(1)	0.65(2)	1.03(2)
5	0.4326(4)	0.219(5)	0.59(1)	0.61(3)	1.03(2)
6	0.4550(6)	0.249(5)	0.54(1)	0.53(1)	1.04(2)
7	0.4699(6)	0.244(5)	0.54(1)	0.53(1)	1.03(2)
8	0.4832(5)	0.261(4)	0.51(1)	0.51(1)	1.04(2)
10	0.5031(9)	0.299(2)	0.45(1)	0.48(1)	1.04(2)
14	0.5282(1)	0.300(3)	0.44(1)	0.45(1)	1.04(1)
20	0.5494(3)	0.307(3)	0.43(1)	0.47(1)	1.05(1)
25	0.5617(1)	0.309(4)	0.43(1)	0.44(1)	1.05(2)
50	0.5918(1)	0.326(4)	0.40(1)	0.41(1)	1.05(1)

**Figure 7:**
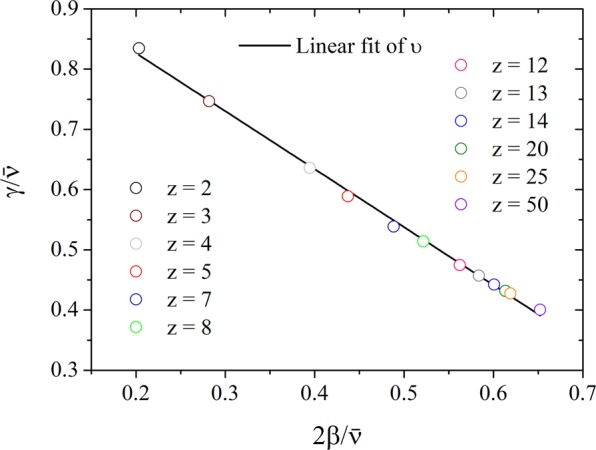
Plot of the characteristic unitary line *y* = −0.96(1)*x* + 1.02(1) estimated by the linear fit of the relation between the critical exponents $\beta /\bar{\nu }$ and $\gamma /\bar{\nu }$ for several values of the growth parameter *z*. The error bars are smaller than the symbol size.

By relating the critical exponents, we obtain the characteristic unitary line of the model showed in Fig. 7. Here, we plot the values of the critical exponents $\gamma /\bar{\nu }$ versus $2\beta /\bar{\nu }$. The linear fit of the data yield $y=-0.96(1)x+1.02(1)$, and an averaged unitary exponent $\upsilon =1.02(1)$ for the three-state majority-vote model on Barabási-Albert networks. We conjecture that this line is universal, regardless of the geometric structure of the network used in the model. Thus, one can use the volumetric scaling *ξ* ~ *N* with the unitary relation Eq. (17), and the Eqs. (18), (19) and (20) to obtain the critical exponents and the unitary line for any spin model under consideration, with or without a system size length clearly defined.

**Figure 8:**
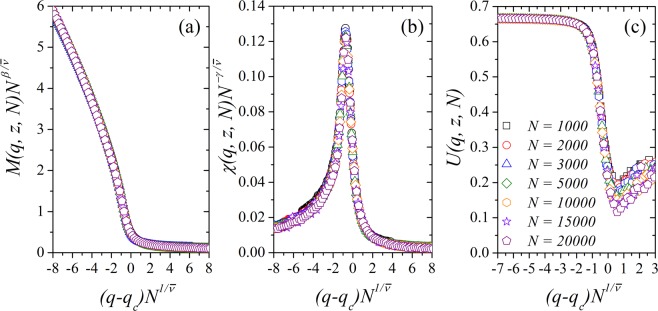
Data collapse for (**a**) the magnetization *M*(*q*, *z*, *N*), (**b**) the magnetic susceptibility *χ*(*q*, *z*, *N*) and (**c**) the Binder’s fourth-order cumulant *U*(*q*, *z*, *N*) for *N* = 1000, 2000, 3000, 5000, 10000, 15000 and 20000 with *z* = 14. Here, we used $\beta /\bar{\nu }=0.3$, $\gamma /\bar{\nu }=0.44$ and $1/\bar{\nu }=0.45$. The error bars are smaller than the symbol size.

Figure 8 shows the plot of the rescaled (a) magnetization $M(q,z,N){N}^{\beta /\bar{\nu }}$, (b) susceptibility $\chi (q,z,N){N}^{-\gamma /\bar{\nu }}$, and (c) Binder cumulant *U*(*q*, *z*, *N*) versus rescaled noise parameter $(q-{q}_{c}){N}^{1/\bar{\nu }}$ for the growth parameter *z* ~ 14. Here, we used $\beta /\bar{\nu }=0.300$, $\gamma /\bar{\nu }=0.44$ and $1/\bar{\nu }=0.45$ with *q*_*c*_ = 0.5282. Other values for the growth parameter $z$ exhibit the same features and the same qualitative results for the data collapse of the magnetization, susceptibility, and Binder cumulant. From our simulation results and analysis, we conclude that the three-state majority-vote model defined on Barabási-Albert networks and on the Erdös–Rényi random graphs belong to different universality classes when the volumetric scaling *ξ* ~ *N* is used^33^.

### Unitary relation on regular networks

**Figure 9:**
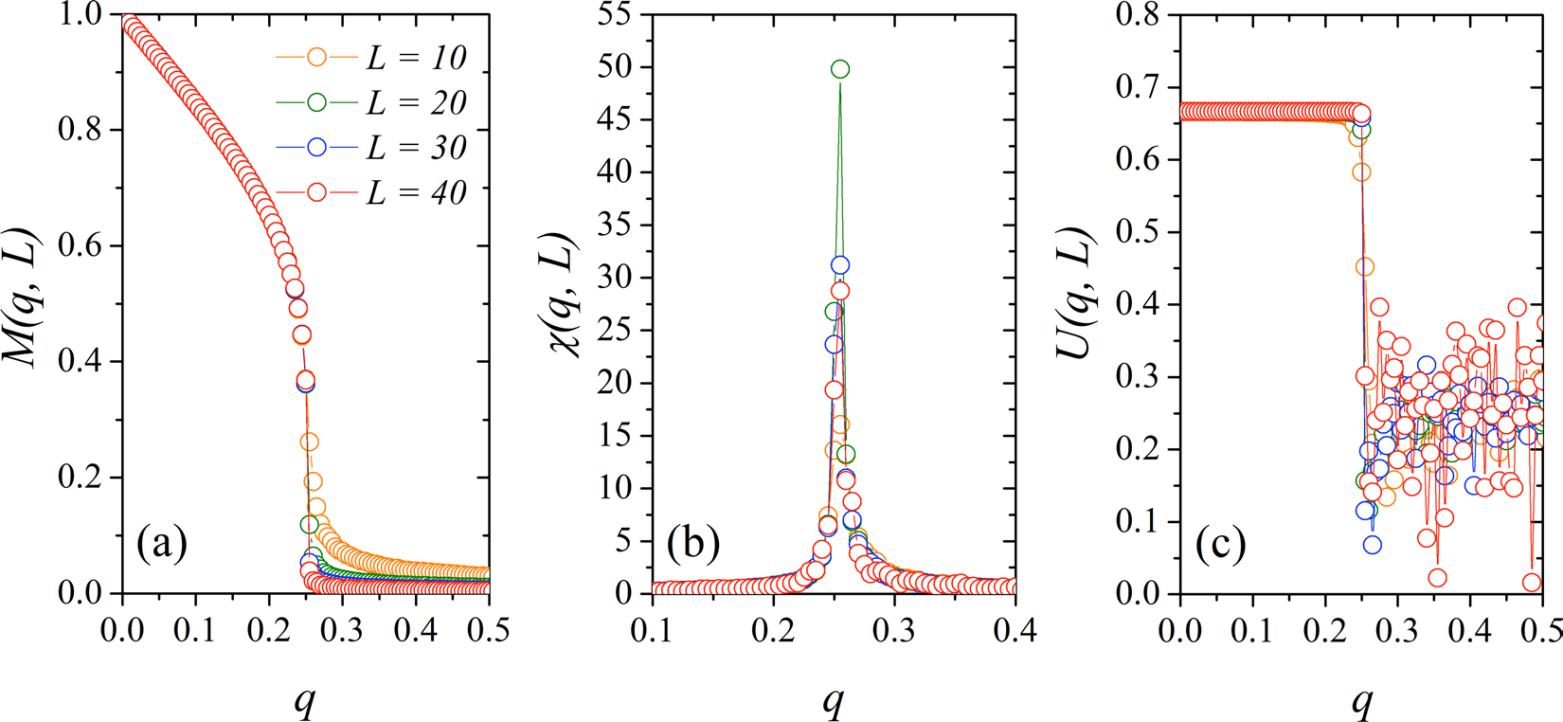
(**a**) Magnetization *M*(*q*, *L*), (**b**) magnetic susceptibility *χ*(*q*, *L*) and (**c**) Binder cumulant *U*(*q*, *L*) for the three-state majority-vote model on cubic networks with *L* = 10, 20, 30 and 40 and periodic boundary conditions. In all plots the error bars are smaller than the symbol size.

To confirm the validity of our statements, we performed Monte Carlo simulations for the majority-vote model with two and three states on regular square lattices and cubic networks. From our simulations of the three-state majority vote model on cubic networks, we obtain the Fig. 9 that shows the (a) magnetization *M*(*q*, *L*), the (b) susceptibility *χ*(*q*, *L*) and the (c) Binder cumulant *U*(*q*, *L*) versus the noise parameter *q*, where *N* = *L*^3^. We observe some familiar results such as *M*(*q*, *L*) → 0 for *q* > *q*_*c*_, with *L* → ∞, and *χ*(*q*, *L*) that also exhibits a sharper peak as we increase *L*. From the Binder parameter, we find the critical noise of the model *q*_*c*_ = 0.25230(2).

By performing numerical simulations for the majority-vote model with two and three states on regular square lattices and on cubic networks, we build the Table 2 with the critical exponents for the magnetization and susceptibility. We also calculate the unitary relation $\upsilon$ and effective dimension *d* obtained for each model with the volumetric (*ξ* ~ *N*) and linear (*ξ* ~ *L*) scalings, respectively. We conclude that the critical exponents with the linear and volumetric scalings relate by $\beta /\nu =d\beta /\bar{\nu }$ and $\gamma /\nu =d\gamma /\bar{\nu }$, as expected by recalling that *ξ* ~ *L*^*d*^.

**Table 2:** The critical noise *q*_*c*_,  the critical exponents with the volumetric scaling *ξ* ~ *L*^*d*^, the unitary relation $\upsilon$, the regular critical exponents *β*/*v* and *γ*/*v*, and the effective dimension *d* when *ξ* ~ *L* for the majority-vote model with two and three states on regular networks.

Network	States (*Q*)	*q*_*c*_	${\boldsymbol{\beta }}{\boldsymbol{/}}\bar{{\boldsymbol{\nu }}}$	${\boldsymbol{\gamma }}/\bar{{\boldsymbol{\nu }}}$	${\boldsymbol{\upsilon }}$	*β*/*v*	*γ*/*v*	*d*
*Square*	3	0.118(1)	0.067(1)	0.90(1)	1.03(1)	0.134(1)	1.80(1)	2.07(1)
*Square*	2	0.075(1)	0.062(1)	0.87(1)	0.99(1)	0.124(1)	1.74(1)	1.99(1)
*Cubic*	3	0.2523(1)	0.197(5)	0.64(1)	1.03(2)	0.33(1)	2.41(1)	3.07(3)
*Cubic*	2	0.1761(3)	0.154(2)	0.70(1)	1.01(1)	0.461(7)	2.11(1)	3.03(2)

**Figure 10:**
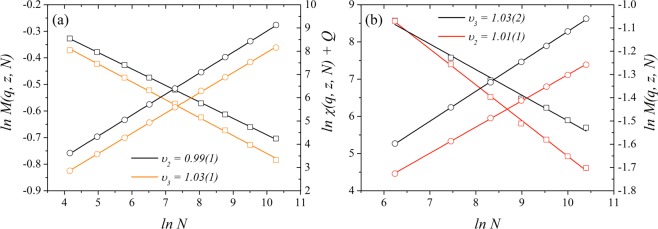
Logarithm of the magnetization (squares) and of the susceptibility (circles) for the majority-vote model with *Q* = 2 and *Q* = 3 states as a function of the logarithm of the system sizes *N* in for (**a**) square lattice and (**b**) cubic networks. We obtain for regular square lattices $\beta /\bar{\nu }=0.067(1)$, $\gamma /\bar{\nu }=0.90(1)$ and ${\upsilon }_{3}=1.03(1)$ for *Q* = 3, and $\beta /\bar{\nu }=0.062(1)$, $\gamma /\bar{\nu }=0.87(1)$ and ${\upsilon }_{2}=0.99(1)$ for *Q* = 2. For a cubic network (**b**), we obtain $\beta /\bar{\nu }=0.107(5)$, $\gamma /\bar{\nu }=0.81(1)$ and ${\upsilon }_{3}=1.02(2)$ for *Q* = 3, and $\beta /\bar{\nu }=0.154(2)$, $\gamma /\bar{\nu }=0.70(1)$ and ${\upsilon }_{2}=1.01(1)$ for *Q* = 2.

In Fig. 10, we plot the logarithm of the magnetization, and magnetic susceptibility used to obtain the critical exponents for the majority-vote model with two and three states on a square lattice and cubic networks, where we used the volumetric scaling. Our results confirm that the unitary relation holds for this model on these networks, and it points that the effective dimension obtained by previous works with the majority-vote model on random graphs and on Barabási-Albert networks might be not equal to unity^25,28,33,38,39^.

## Conclusion and Final Remarks

We have investigated the dynamics of the three-state majority-vote model for opinion dynamics on Barabási-Albert networks. We obtained the phase diagram and the critical exponents of the model, and we verified that the second-order phase transition occurs for networks with growth parameter *z* > 1. The critical exponents $\gamma /\bar{\nu }$ and $\mathrm{1/}\bar{\nu }$ decrease with *z*, while $\beta /\bar{\nu }$ increases. We also find that the critical noise *q*_*c*_ is an increasing function of the growth parameter *z*, which converges to the infinite social temperature value 2/3 as *z* → ∞. In other words, if *z* is big enough, the three-state opinion society remains ordered, with a consensus, even for a high value of the social disorder (or preference for dissensus *q*). From Fig. 5 we estimate that this occurs for *z* ≥ 20, where *q*_*c*_ ~ 2/3, indicating that it is harder to destroy consensus in a more connected society.

By assuming that near criticality, the correlation length *ξ* scales with the actual volume of the system *ξ* ~ *N*, we found that the new hyperscaling relation is equal to 1, regardless of the effective dimension of the network of interactions. Nevertheless, by performing Monte Carlo simulations we verified that the unitary relation $\upsilon \equiv 2\beta /\bar{\nu }+\gamma /\bar{\nu }=1$ for all values of the growth parameter *z* investigated. The unitary relation was also verified for the majority-vote model with two and three states on regular square lattices and cubic networks, with well defined effective dimensions. Our analysis sheds light on a curious result of several works, where authors find that the effective dimension of different complex networks is equal to one^25,28,33,38,39^. We show that this result is a consequence of the scaling used and that this is not the real dimension of those networks, excluding eventual coincidences. Furthermore, the unitary relation here defined for a sociophysics model holds for other spin-like models in condensed matter systems, regarding the geometric dimensions in which these systems are embedded.

We remark that obtaining the effective dimension for some complex networks - such as the random graphs and the Barabási-Albert networks - using the finite-size scaling analysis near the critical points of such spin systems, and the relations between its critical exponents, remains a task to perform. Although other methods and scalings have been successfully developed^46,47^. Our results may suggest future research finding and using other finite-size scaling relations, such as some power-law with a characteristic length instead. We also recommend the use of the unitary relation (Eq. (17)) and the unitary line (Fig. 7), defined and discussed in this work, as a form investigation of the criticality of systems with complex interactions of unknown effective dimension, or with a linear size not precisely defined.
